# Serum Syndecan-1 Levels and Its Relationship to Disease Activity in Patients with Crohn's Disease

**DOI:** 10.1155/2015/850351

**Published:** 2015-07-29

**Authors:** Cem Çekiç, Adnan Kırcı, Sezgin Vatansever, Fatih Aslan, Huriye Erbak Yılmaz, Emrah Alper, Mahmut Arabul, Elif Sarıtaş Yüksel, Belkıs Ünsal

**Affiliations:** ^1^Department of Gastroenterology, Katip Çelebi University, Atatürk Training and Research Hospital, 35360 İzmir, Turkey; ^2^Department of Gastroenterology, Faculty of Medicine, Şifa University, 35100 İzmir, Turkey; ^3^Department of Biochemistry, Katip Çelebi University, Atatürk Training and Research Hospital, 35360 İzmir, Turkey

## Abstract

*Background*. Syndecan-1 (SDC-1), a member of the family of heparan sulfate proteoglycans, plays an important role in the resolution of inflammation. This study aimed to investigate the relationship between SDC-1 and disease activity in Crohn's disease (CD). *Methods*. Serum samples of 54 patients with CD and 30 healthy controls were obtained. First, SDC-1 levels of the CD patients were compared to the control group. Subsequently, SDC-1 levels were analyzed in patients with CD in active and remission periods. Finally, SDC-1 efficacy in predicting disease activity was evaluated by performing correlation analysis between SDC-1 and C-reactive protein (CRP) and Crohn's disease activity index (CDAI). *Results*. SDC-1 level was higher in the CD group (61.9 ± 42.6 ng/mL) compared with the control group (34.1 ± 8.0 ng/mL) (*p* = 0.03). SDC-1 levels were higher in active CD patients (97.1 ± 40.3 ng/mL) compared with those in remission (33.7 ± 13.5 ng/mL) (*p* < 0.001). A significant positive correlation was found between SDC-1 and CRP (*r* = 0.687, *p* < 0.001) and between SDC-1 and CDAI (*r* = 0.747, *p* < 0.001). *Conclusion*. Serum levels of SDC-1 are higher in CD compared to the normal population and can be an effective marker of disease severity.

## 1. Introduction

Crohn's disease (CD) is a chronic idiopathic inflammatory disease. Although the exact cause is unknown, a complex interaction of environmental, genetic, and immunologic factors is held responsible for the pathogenesis of the disease [1]. To date, although markers such as fecal calprotectin and C-reactive protein (CRP) are widely used in the determination of the disease activity in CD, methods to measure disease progression and mucosal recovery are still being studied [2].

Syndecan-1 (SDC-1) is a type 1 transmembrane heparan sulfate proteoglycans. The majority of HSPGs are expressed by the epithelial cells and plasma cells. SDC-1 is composed of covalently linked glycosaminoglycan chains to a core protein. There are three different domains of SDC-1 (cytoplasmic, transmembrane, and extracellular) which exist in organisms [3, 4].

Although the main function of SDC-1 in adult mammals is the resolution of inflammation, it also has an important role in the pathogenesis of wound healing, fibrosis, tumor biology, and infectious diseases [5].

SDC-1, located on the cell membrane, serves as a coreceptor for many extracellular ligands including primary inflammatory cytokines and growth factors. Therefore, SDC-1 plays a key role in many metabolic processes which have a role in maintaining hemostasis and cellular morphology [6].

Syndecans in the cell surface can be shed by a variety of matrix proteinases during inflammatory processes. Soluble SDC-1 increases with the shedding [7].

SDC-1 deficiency has been shown to play a role in the development of many acute and chronic diseases with uncontrolled and long-term inflammation in animal studies and in vitro models [8].

SDC-1 has a pivotal role in preserving the intestinal barrier function and preventing bacterial translocation working synergistically with tight junctions through Stat3 signaling [9]. Moreover, the involvement of SDC-1 in fundamental processes associated with inflammatory bowel disease (IBD) such as regulation of fibrosis, control of inflammation, and wound healing and the pathogenesis and disease progression of IBD has not been fully identified.

In this study, we aimed to determine the serum levels of SDC-1 and to evaluate the relationship between serum levels of SDC-1 and disease activity in Crohn's disease.

## 2. Patients and Methods

### 2.1. Patient Selection

Thirty healthy controls and 54 patients with CD aged 18 years or older, who were followed by the IBD unit of Department of Gastroenterology of Izmir Katip Çelebi University, were enrolled in the study.

### 2.2. Study Design

The demographic data of the healthy controls and patients with CD included in the study were obtained. Disease localization, behavior types, and treatment modalities related to CD were recorded. The Montreal classification was used to determine the location and behavior patterns of CD [10]. Crohn's disease activity index (CDAI) was used to determine disease activity (CDAI < 150 remission, CDAI ≥ 150 active) [11]. Blood samples were obtained from patients concurrently for the measurement of serum SDC-1 and CRP. After comparing the serum levels of SDC-1 of the patient and control groups, patients with CD were divided into two groups: patients in active period and patients in remission period according to the CDAI. Subsequently, serum levels of SDC-1 of the patients with CD in the active and remission periods were compared. Further, a correlation analysis between serum SDC-1, CRP, and CDAI was performed.

### 2.3. Exclusion Criteria

Patients with solid organ or hematologic malignancy, with an active infection, receiving low molecular weight heparin, with angiotensin II receptor antagonist use, and with insulin-treated diabetics and patients with cardiac, hepatic, or renal failure and pregnant woman were excluded from the study.

### 2.4. Assessment of Serum SDC-1 Levels

Eight–10 mL of venous blood was drawn from patients with Crohn's disease and healthy controls enrolled in the study. The serum was centrifuged at 4,000 rpm for 20 min in sterile conditions. Sera were stored in clean and dry microcentrifuge tubes at −20°C prior to analysis. Patient serum and a standard solution were pipetted into human SDC-1 antibody coated wells. Biotin-conjugated anti-CD 138 antibody was added to each well. After incubation at room temperature (18–25°C) for an hour, the wells were washed three times with 300 microliters of wash solution. Next, Streptavidin-HRP solution was added and allowed to incubate at room temperature for 30 min, and then the washing procedure was repeated. Chromogen solution was added and incubation was carried out in the dark and the reaction was terminated with H_2_SO_4_. Concentration was calculated according to a standard absorbance curve after absorbance was read at 450 nm by an ELISA plate reader. Human SDC-1/CD138 ELISA kit (Diaclone, Besancon Cedex, France, reference number: 950 640 096, lot #0138-42) was used with a Biotek semiautomatic ELISA processor.

### 2.5. Statistical Analysis

All statistical analyses were performed using SPSS 18.0 statistical package program. Continuous variables were described by means, standard deviations, medians, and min-max values, and categorical variables were described by frequencies and percentages. Chi square and Fisher's exact test were used to compare two categorical variables. The Kruskal Wallis test was used to compare more than two means. The differences between two independent samples' means or medians were compared by Mann-Whitney* U* test or median test. The correlations of SDC-1 with CRP and CDAI were tested by Spearman correlation analysis. ROC analyses were performed to understand the impacts of SDC-1 and CRP on disease activity. The Youden Index method was used for cut-off analysis of SDC-1. We ran power analyses using GPower3 statistical software. The achieved power (1 − *β*) was 0.99 for the comparison of SDC-1 levels between the patient and the control group. *p* < 0.05 was accepted as statistically significant.

## 3. Ethical Considerations

The Ethics Committee of Katip Celebi University Faculty of Medicine, Izmir, Turkey, approved this study. Written informed consent was obtained from each patient prior to study.

## 4. Results

### 4.1. Patient Characteristics

In this study, we evaluated 54 patients with CD and 30 healthy controls. While the mean age of the patients with CD was 39.3 ± 12.2, the mean age of the control group was 38.9 ± 11.8. Twenty-eight (51.9%) CD patients and 15 (50%) control group patients were male. No statistically significant difference was observed between the CD group and the control group in terms of age and gender (*p* = 0.765 and *p* = 0.871, resp.). Demographic and clinical characteristics of the patients with CD are presented in Table 1.

### 4.2. Factors Effecting the Syndecan-1 Levels in Crohn's Disease

**Figure 1:**
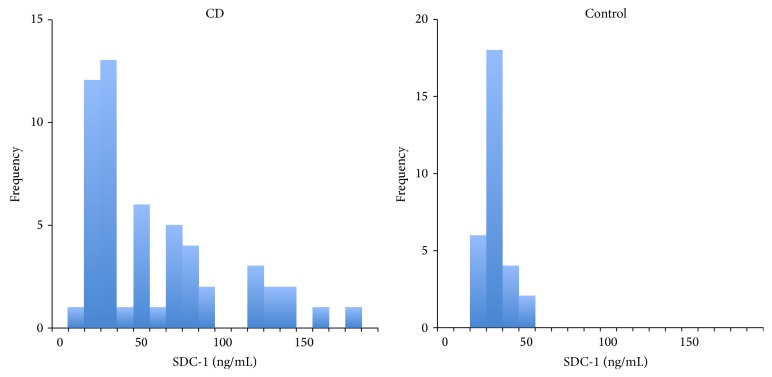
Histograms of serum SDC-1 levels of the patients with Crohn's disease and control group.

Serum SDC-1 level was found to be significantly higher in the CD group (61.9 ± 42.6 ng/mL) when compared to the control group (34.1 ± 8.0 ng/mL) (Figure 1).

Factors, such as disease activity, CD subtype, the presence of perianal disease, treatment methods, and steroid requirement, all of which may have an impact on serum SDC-1 levels, were seen to create significant differences on serum SDC-1 levels in the univariate analysis in the CD group. No significant effect of CD localization was detected on serum SDC-1 level (Table 2). No significant relationship was found between the age and the serum SDC-1 levels in the correlation analysis carried out between age and SDC-1 levels (*r* = −0.168, *p* = 0.226). Serum SDC-1 levels between male and female genders were not found to be statistically different (*p* = 0.272) (68.4 ± 36.2 ng/mL, 52.6 ± 27.4 ng/mL, resp.).

Serum SDC-1 levels were found to be statistically significantly different between the penetrating and stricturing disease in post hoc analysis done for CD subtypes (*p* = 0.026). In other pairwise comparisons, there was no statistically significant difference (*p* > 0.05).

The differences between the serum SDC-1 levels of the patients receiving 5-aminosalicylic acid (5-ASA) versus antitumor necrosis factor (anti-TNF) (*p* = 0.009) and the patients receiving Azathioprine (AZA) versus anti-TNF patients (*p* = 0.027) were found to be statistically significant in post hoc analysis done for the treatment methods. In other pairwise comparisons there was no statistical significance (*p* > 0.05). The ASA, AZA, anti-TNF, and AZA + anti-TNF groups had the CDAI scores as follows: 111.5 ± 44.0, 139.4 ± 64.9, 174.0 ± 42.8, and 158.8 ± 64.5, respectively (*p* < 0.05). In the post hoc analysis, the difference originated from means of CDAI scores of ASA and anti-TNF (*p* = 0.012). The rest of the pairs did not reveal a statistically significant difference.

### 4.3. Serum Syndecan-1 and Detection of Disease Activity

Serum SDC-1 and CRP levels were found to be significantly higher in the patients with active CD compared to remission (*p* < 0.001). CDAI scores and serum SDC-1 and C-reactive protein levels in active and remission periods are listed in Table 3.

A strong positive correlation was found between SDC-1 and CRP (*r* = 0.687, *p* < 0.001) and the SDC-1 and CDAI (*r* = 0.747, *p* < 0.001) in a Pearson correlation analysis conducted between SDC-1 and CRP and CDAI.

**Figure 2:**
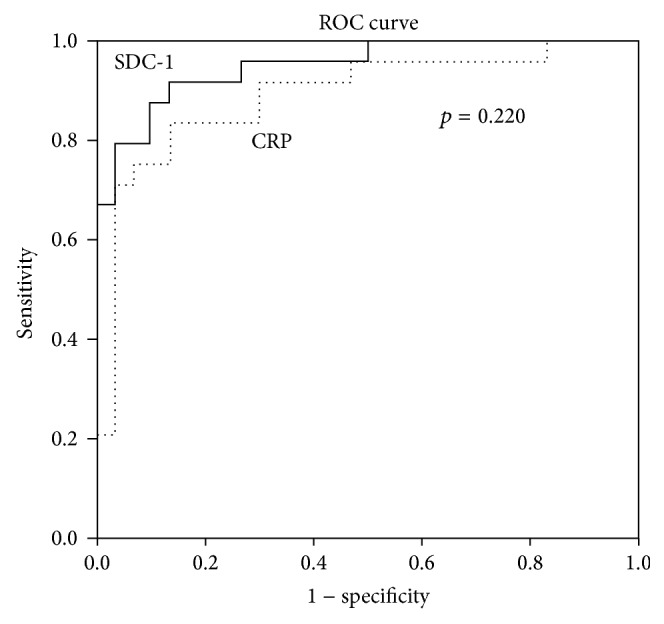
Efficiency of SDC-1 and CRP levels for the assessment of disease activity.

Both SDC-1 and CRP were found to be quite effective in detecting disease activity in ROC analysis. The areas under the curves were equal (*p* = 0.220) (Figure 2).

52.5 ng/mL was determined as the cut-off value for SDC-1 by the Youden Index method. The detection sensitivity of disease activity with this cut-off level was 91.7%, the specificity 86.7%. Active disease risk was found to be 71.5 times higher in patients with SDC-1 level ≥52.5 ng/mL compared to patients below this value (OR = 71.5, 95% CI: 11.9–428.2).

## 5. Discussion

Inappropriate inflammatory response and impaired intestinal mucosal integrity are the basic pathologies that play a role in the pathogenesis of Crohn's disease (CD) [12]. Determination of the role of SDC-1, an important protein in the regulation of inflammation, control of wound healing, and protection of epithelial integrity, in the pathogenesis, diagnosis, and the follow-up of inflammatory bowel diseases (IBD) would be useful.

Serum SDC-1 levels, the factors affecting the SDC-1 levels, and the role of SDC-1 in predicting disease activity in CD were investigated in this study.

In our study, mean serum SDC-1 levels were significantly higher in CD patients compared to healthy controls (*p* = 0.03). In the majority of experimental colitis models and human studies conducted to date, mucosal SDC-1 levels were shown to be lower and serum SDC-1 levels higher in IBD compared to the normal population [13–15]. (Serum SDC-1 is also defined as soluble SDC.) Increased serum SDC-1 levels have been shown to be related to shedding and destruction of mucosal SDC-1 caused by inflammatory cytokines or severe inflammation in these studies. Thus, increased serum SDC-1 levels can be considered an indirect indicator of decreased mucosal SDC-1. In a study by Zhang et al., similarly to our study, serum SDC-1 levels were found higher in CD compared to patients with functional bowel disease or intestinal tuberculosis [16]. On the other hand, IBD patients had higher soluble SDC-1 levels compared to healthy controls in the research by Yablecovitch et al. However, in the same study, no correlation was found in SDC-1 between CRP and disease activity in contrast to our study. The reason for this difference may be attributed to the presence of large group of UC patients which has no significant difference in SDC-1 levels with the healthy controls in the subgroup analysis [17].

The disease activity, CD subtype, presence of perianal disease, treatment methods, and steroid requirement were all detected to generate a significant difference; however, CD localization, age, and gender were found not to have a significant effect on serum SDC-1 levels in patients with CD in this study.

Severe disease activity, perianal disease, and steroid requirement parameters can be considered together as components leading to a common denominator of severe inflammation or adverse clinical outcomes. Increased inflammatory cytokine burden can be regarded as a factor leading to enhanced destruction of the mucosal SDC-1 thereby leading to elevated serum SDC-1 levels in patients with mentioned adverse factors. The mucosal SDC-1 was shown to decrease and serum SDC-1 levels were shown to increase in parallel with the severity of histopathological damage in IBD mostly in experimental colitis models [18].

Low serum SDC-1 levels particularly in stricturing CD were noteworthy in a post hoc analysis done for CD subtypes. SDC-1 plays an important role in the regulation of the factors, mainly basic fibroblast growth factor (bFGF), that have a part in the fibrogenesis process [19]. In addition, SDC-1 is an effective factor in the fibroblast proliferation and fibrosis development via transforming growth factor-beta (TGF-*β*) [5]. Ierardi et al. detected increased bFGF and SDC-1 levels in the resection specimens from CD patients with surgical resection secondary to fibrotic strictures in their study [20]. In the light of these data, low serum levels of SDC-1 in stricturing CD can be regarded as indirect evidence of less shedding of mucosal SDC-1 and higher levels of mucosal SDC-1 in our study.

Serum SDC-1 levels were found to be higher in CD patients receiving anti-TNF treatment. An inverse relationship was shown between mucosal SDC-1 and TNF-*α* levels in CD. The inhibition of the expression of SDC-1 by TNF-*α* was shown to be the main cause of this condition in the past studies [21]. Breaking of this inhibition with anti-TNF may explain the increase in SDC-1 expression in anti-TNF treatment group. On the other hand, severe inflammation and mucosal damage in CD patients under immunomodulatory or biologic treatment may cause higher serum levels of SDC-1.

In the study of Zhang et al., SDC-1 was shown to be an effective marker in determining disease activity in CD [16]. A strong positive correlation was found to be between SDC-1 and CRP and CDAI in our study, too. Moreover, SDC-1 is considered to be as effective as CRP in the prediction of disease activity.

Our study has some limitations. Determination of mucosal SDC-1 expression concurrently with serum SDC-1 levels and detection of the severity of mucosal disease endoscopically, in addition to CDAI, may have increased the power of our study. Additionally, we believe that the determination of the serum levels of cytokines, such as TNF-*α* and interleukin-1*β*, which are effective in shedding and expression of SDC-1 and are major inflammatory cytokines of CD, will provide an additional contribution to future studies on this subject.

In conclusion, serum SDC-1 levels are higher in CD patients than the normal population. Also, serum SDC-1 levels appear to be an effective marker of disease severity. Therefore, in addition to daily practice determination of serum levels of SDC-1 may contribute to the identification of disease activity in clinically intermediate cases.

## Figures and Tables

**Table 1:** Demographics and baseline characteristics.

	Total (*n* = 54)		Active (*n* = 24)		Remission (*n* = 30)		*p*
Age (mean, SD)	39.3	12.2	38.6	13.6	39.8	11.2	0.774
Gender (*n*, %)							
Male	28	51.9	14	58.3	14	46.7	0.394
Female	26	48.1	10	41.7	16	53.3	
Disease duration (years) (median, min–max)	3.5	1–16	4.0	1–16	3.0	1–14	0.055
Localization (*n*, %)							
Colonic	8	14.8	4	16.7	4	13.3	NS
Ileal	18	33.3	6	25	12	40	
Ileocolonic	28	51.9	14	58.3	14	46.7	
CD behavior (*n*, %)							
Penetrating	9	16.7	6	25	3	10	0.083
Stricturing	22	40.7	6	25	16	53.3	
Inflammatory	23	42.6	12	50	11	36.7	
CDAI (mean, SD)	139.1	58.1	193.0	40.5	96.1	23.5	<0.001

SD, standard deviation; CD, Crohn's disease; NS, nonsignificant; CDAI, Crohn's disease activity index.

**Table 2:** The effects of disease characteristics and the treatment methods on serum SDC-1 level.

	*N*	Mean	SD	*p*
Disease activity				
Active	24	97.1	40.3	<0.001
Remission	30	33.7	13.5	
Localization^a^				
Colonic	8	68.6	42.5	0.240
Ileal	18	49.5	35.9	
Ileocolonic	28	67.9	46.2	
CD behavior^b^				
Penetrating	9	89.9	51.8	0.048
Stricturing	22	51.4	41.4	
Inflammatory	23	61.0	36.5	
Perianal disease				
Yes	8	95.1	50.9	0.034
No	46	56.1	38.8	
Treatment^c^				
5-ASA	17	42.8	20.3	0.027
AZA	23	54.8	36.3	
Anti-TNF	8	108.3	57.2	
AZA + anti-TNF	6	81.6	47.0	
Steroid requirement				
Yes	36	73.5	46.3	0.004
No	18	38.7	20.0	

SD, standard deviation; CD, Crohn's disease; ASA, aminosalicylic acid; AZA, Azathioprine; TNF, tumor necrosis factor.

a: colonic versus ileal (*p* = 0.243); colonic versus ileocolonic (*p* = 0.924); ileal versus ileocolonic (*p* = 0.110)

b: penetrating versus stricturing (*p* = 0.026); penetrating versus inflammatory (*p* = 0.137); stricturing versus inflammatory (*p* = 0.131).

c: ASA versus AZA (*p* = 0.412); ASA versus anti-TNF (*p* = 0.009); ASA versus AZA + anti-TNF (*p* = 0.093); AZA versus anti-TNF (*p* = 0.027); AZA versus AZA + anti-TNF (*p* = 0.146); anti-TNF versus AZA + anti-TNF (*p* = 0.302).

**Table 3:** Comparison of serum SDC-1 and CRP levels according to the disease activity.

	Active		Remission		*p*
	Mean	SD	Mean	SD	
SDC-1 (ng/mL)	97.1	40.3	33.7	13.5	<0.001
CRP (mg/dL)	4.3	4.2	0.7	1.3	<0.001
CDAI	193.0	40.5	96.1	23.5	<0.001

SDC-1, Syndecan-1; CRP, C-reactive protein; CDAI, Crohn's disease activity index.

## Abbreviations

ASA: Aminosalicylic acid
AZA: Azathioprine
CD: Crohn's disease
CDAI: Crohn's disease activity index
CRP: C-reactive protein
IBD: Inflammatory bowel disease
SDC-1: Syndecan-1
TNF: Tumor necrosis factor.
